# The kebab technique uses a bipolar pencil to retrieve a dropped nucleus of the lens via a small incision

**DOI:** 10.1038/s41598-021-87022-3

**Published:** 2021-04-12

**Authors:** Hiroshi Aso, Harumasa Yokota, Hirotsugu Hanazaki, Satoru Yamagami, Taiji Nagaoka

**Affiliations:** Division of Ophthalmology, Department of Visual Sciences, Nihon University School of Medicine, 30-1 Oyaguchi-Kamicho, Itabashi-ku, Tokyo, 173-8610 Japan

**Keywords:** Lens diseases, Vision disorders

## Abstract

We developed a new method to retrieve a dropped nucleus of the lens via a small incision using bipolar pencils, the kebab technique, to solve the lack of small-gauge fragmatomes, and the expense and toxicity of perfluorocarbon liquids (PFCL). A total of 8 eyes in 6 patients underwent this technique and were reviewed. After vitrectomy, the dropped nucleus of the lens was lifted from the retina by adhesion with a bipolar pencil, and phacoemulsification was performed while rotating the lens. The outcome measures were best-corrected visual acuity (BCVA), intraocular pressure (IOP), and corneal endothelial cell density before and after surgery. Surgical indications included zonular weakness, trauma, acute angle closure attack, and phacolytic glaucoma. At 1 month, BCVA improved from a mean (standard deviation, SD) 1.67 logMAR (0.90) to 1.14 logMAR (1.01). The mean preoperative IOP was 24.5 (16.8) mmHg and postoperative IOP was 11.0 (2.8) mmHg. The mean preoperative corneal endothelial cell count was 2600 (322) cells/mm^2^ (one eye was unmeasurable) and postoperative corneal endothelial cell count was 2387 (431) cells/mm^2^. There were no postoperative complications. The retrieval of a dropped nucleus of the lens using a bipolar pencil enables small incisions without using PFCL.

## Introduction

Although recent advances in phacoemulsification apparatus have been improving surgical performance, the dropped nucleus of the lens is one of several intraoperative complications during cataract surgery that needs to be addressed. To preserve vision, the dropped nucleus of the lens must be removed while minimizing damage to the cornea, iris, and retina.

A fragmatome was previously used to emulsify and remove a nucleus in the vitreous with 20- and 23-gauge systems^1^. However, in the era of micro-incision vitrectomy surgery (MIVS) with 25- and 27-gauge systems, this device is too large to be used. Small fragments of the dropped nucleus of the lens can be safely removed with a small-gauge vitreous cutter, but it takes a long time to remove larger fragments because it is difficult to fix them, which risks damage to the macula.

Instead of these techniques, perfluorocarbon liquids (PFCL) are often used to float the dropped nucleus of the lens because of the advantages of avoiding retinal tearing and protecting the macula from fragments of the nucleus^2–4^. When the vitreous is filled with PFCL, the falling lens will rise to the iris plane, allowing the safe performance of phacoemulsification. However, PFCL is costly and residual PFCL in the vitreous may cause adverse effects^5,6^.

We have developed a new technique of lifting and fixing the dropped nucleus of the lens without using PFCL called the “kebab technique.” This technique enables the operator to lift and fixed the dropped lens and to perform phacoemulsification safely in the anterior chamber or near the anterior vitreous in a method akin to shaving a rotating doner kebab.

## Results

The characteristics of participants and postoperative outcomes are shown in Table 1. The study comprised 8 eyes of 6 patients (3 males and 3 females). The grades of nuclear sclerosis were grade 5 in 2 eyes, grade 4 in 3 eyes, and grade 3 in 3 eyes. The mean age at the time of surgery was 77.9 (4.11) y. The indications for lens luxation or subluxation were zonular weakness in three eyes, trauma in two eyes, phacolytic glaucoma in two eyes, and acute angle closure attack in one eye. In six eyes, IOL implantation was performed with the Yamane double-needle intrascleral lens fixation technique. We inserted an AN6KA (Kowa, Tokyo, Japan) into four eyes and an X-70 (Santen, Osaka, Japan) into two eyes. In the two cases of phacolytic glaucoma, only lens removal was performed and IOL intrascleral fixation was not performed. In all eight consecutive cases, the bipolar pencil was firmly attached to the center of the nucleus by surface coagulation, allowing safe phacoemulsification.

**Table 1:** The characteristics of participants and postoperative outcomes.

	Mean ± SD 8 eyes of 6 patients
Age (years)	77.9 ± 4.11
Sex	Males (n = 3) Females (n = 3)
NS^a^	3.88 ± 0.78
Axial Length	23.13 ± 0.40
**BCVA**^**b**^** (logMAR)**^**c**^	
Preop	1.67 ± 0.90
Postop at 1 month	1.14 ± 1.01
**IOP**^**d**^** (mmHg)**	
Preop	24.5 ± 16.8
Postop at 1 month	11.0 ± 2.8
**Corneal endothelial cell density (cells/mm**^**2**^**)**	
Preop	2600 ± 323
Postop 1 month	2387 ± 431

^a^NS: Nuclear Sclerosis.

^b^BCVA: best-corrected visual acuity.

^c^logMAR: logarithm of the minimum angle of resolution.

^d^IOP: intraocular pressure.

Individual patient data are provided in Table 2. The mean axial length was 23.13 (0.38) mm. BCVA improved from mean 1.67 logMAR (0.90) to 1.14 logMAR (1.01) at 1 month after surgery. The mean (SD) preoperative intraocular pressure (IOP) was 24.5 (16.8) mmHg and postoperative IOP was 11.0 (2.8) mmHg. The mean preoperative corneal endothelial cell density was 2600 (322) cells/mm^2^ (one eye was unmeasurable) and the postoperative corneal endothelial cell density was 2387 (431) cells/mm^2^. No postoperative complications such as leakage, postoperative high or low intraocular pressure, cystoid macular edema, vitreous hemorrhage, or retinal detachment occurred.

**Table 2:** Individual patient data.

Case	Age/Sex	Indication	NS^d^	Axial length (mm)	IOL^e^	BCVA^a^ (logMAR^b^)		IOP^c^ (mmHg)		Endothelial cell density (cells/mm^2^)	
						Preop	Postop (1 Month)	Preop	Postop (1 Month)	Preop	Postop (1 Month)
1	77/F	Phacolytic glaucoma	4	23.77	None	2.90	2.90	60	9	2494	1873
2	78/M	Acute angle closure attack	3	22.51	AN6KA	0.22	0.00	28	16	2110	2288
3	74/M	Trauma	3	23.31	X-70	1.10	1.40	14	8	2825	3012
4	74/M	Trauma	3	23.22	X-70	1.40	2.00	12	10	3205	2370
5	72/F	Phacolytic glaucoma	5	23.44	None	2.80	2.00	42	15	Unmeasurable	1613
6	83/F	Zonular weakness	4	22.93	AN6KA	1.85	0.15	14	9	2410	2695
7	83/F	Zonular weakness	4	22.62	AN6KA	0.80	0.30	17	12	2469	2545
8	82/M	Zonular weakness	5	23.21	AN6KA	2.30	0.40	9	9	2688	2703

^a^BCVA: best-corrected visual acuity.

^b^logMAR: logarithm of the minimum angle of resolution.

^c^IOP: intraocular pressure.

^d^NS: Nuclear Sclerosis.

^e^IOL: Intraocular Lens.

## Discussion

Currently, MIVS is mainly used for vitrectomy for retained lens fragments^7^. It is performed for dropped nucleus of the lens with a small-gauge trocar through a small incision^8^. A vitreous cutter can remove a dropped nucleus of the lens and its fragments with a low cut rate^7^. However, when processing a hard nucleus, it is difficult to remove them only with a small-gauge vitreous cutter^9^. Other methods for retrieving a dropped nucleus of the lens have been reported by different operators. A fragmatome has been used to emulsify and remove a dropped nucleus of the lens in the vitreous, but it requires a special handpiece, and cannot be inserted through a small-gauge trocar, such as a 25-gauge or 27-gauge system. In previous reports, the incidence of postoperative retinal detachment after retained lens removal with a fragmatome combined with a 20-gauge system was 4%^10^. Agarwal et al*.*^11^ and Chiang et al.^12^ performed a new levitating technique using a sleeveless phacotip. This technique has the advantage of not requiring special equipment such as a fragmatome, but requires a large 20-gauge pars plana wound. In addition, postoperative retinal cystoid macular edema (11%) has also been reported with this method^12^.

PFCL can be instilled to levitate and remove a dropped nucleus of the lens by phacoemulsification^2–4^. However, in addition to high costs, the residual PFCL has also corneal and retinal toxicities^5,6^. It is difficult to remove all residual PFCL globules from the vitreous. Subretinal PFCL is more difficult to remove^13,14^. Therefore, the kebab technique has the advantage of allowing phacoemulsification without PFCL in the anterior chamber. By bonding the tip of the bipolar pencil to the center of the dropped nucleus of the lens, the lens can be reliably lifted and can undergo phacoemulsification in a safe position.

We initially tried needles and forceps to lift the dropped nucleus of the lens. There was a risk that the needle would damage the retina. Forceps with a blunt tip reduce the risk of serious damage to the retina and allow safe penetration. However, it is difficult to synchronize the hand with the penetrated nucleus using a blunt tip and forceps because the contact between the lens nucleus and the shaft of the forceps is not sufficient to fix the lens. Therefore, we developed this method of bonding the shaft and the nucleus with the aid of a bipolar pencil.

The advantage of this method using a bipolar pencil is that the tip of the bipolar pencil and the nucleus of the lens can be completely bonded by coagulation. Once the bipolar tip reaches the surface of the lens, the entire lens can be lifted either with or without the capsular bag. Thereby, the lifted nucleus can undergo phacoemulsification with its rotation completely controlled. Since the nucleus is emulsified at a position away from the cornea and retina, even with severe nuclear sclerosis as in our cases, there were no complications. In addition, we can perform this technique even in cases of poor mydriasis with a small incision. Therefore, it is possible to remove the nucleus and affix the IOL without any suturing by applying the Yamane double-needle technique^15^. The disadvantage of the kebab method is that some nuclear fragments may fall into the vitreous. By performing phacoemulsification like shaving doner kebab, the lifted nucleus can be removed without dropping a large fragment into the vitreous. The small dropped nuclear fragments can be easily removed with a low-cut-rate vitreous cutter in cases where the nuclear grade is low. If the patients have a hard nucleus (grade 4–5), the nuclear fragments can be completely removed using the kebab technique.

In conclusion, we introduce a new method, the so-called “kebab technique,” to retrieve a dropped nucleus of the lens without using PFCL, through a small incision. Further studies with a larger number of patients will be required to confirm the improvement in safety and optimize our procedure.

## Methods

The study protocol, approved by the Institutional Review Board of the Nihon University School of Medicine (approval number: RK-190611-1), adhered to the tenets of the Declaration of Helsinki. Written informed consent was obtained from all participants. All surgeries were performed by a single surgeon (AH) at the Division of Ophthalmology, Department of Visual Sciences, Nihon University School of Medicine between January 2018 and March 2019. We reviewed cases, mostly of trauma, where the nucleus had been dropped before surgery, as well as cases of lens luxation/subluxation where the nucleus dropped during surgery. All patients underwent a standard ophthalmologic examination, including best-corrected visual acuity (BCVA), slit lamp examination, measurement of intraocular pressure (IOP), and corneal endothelial cell density before and after surgery. Progression of nuclear sclerosis was based on the Emery-Little classification^16^, which was graded by another ophthalmologist (TN) blinded to the type of surgery. BCVA was converted from the Landolt chart at 5 m to the logarithm of the minimum angle of resolution (logMAR)^17^. Counting fingers (CF) was defined as 1.85 logMAR, hand motion (HM) as 2.3 logMAR^18^, light perception (LP) as 2.8 logMAR, and no light perception (NLP) as 2.9 logMAR^19^. The patients were followed up for more than 2 months after the operation. The evaluation before and 1 month after the operation included an ophthalmological examination and assessment of complications.

Under peribulbar anesthesia, a 25-gauge pars plana vitrectomy was performed using a Constellation Vision System (Alcon Laboratories, Inc., Fort Worth, TX). Resight non-contact wide-angle lenses (Carl Zeiss Meditec AG, Jena, Germany) were used to observe the fundus. Vitrectomy was performed from anterior to posterior, and the vitreous around the dropped nucleus of the lens was removed. Then, to confirm the presence or absence of retinal detachment we observed the retinal periphery through wide-angle lenses during scleral depression. A 2.4-mm incision for phacoemulsification and aspiration (PEA) was made at the 12 to 1 o’clock position after anterior and posterior vitrectomy. The phaco setting was set to low vacuum (70 mmHg) and moderate perfusion rate (30 cc/min), and the phaco power was varied from moderate to high depending on the degree of nuclear sclerosis. We used a bipolar pencil (SR-14-5000-10, Kirwan, Marshfield, MA) introduced via the trocar cannula system to fix the dropped nucleus of the lens by surface coagulation and to lift it into the anterior chamber. The diathermic power was set to approximately 50%, which seems to be very high power in vitrectomy. However, this method does not have any influences on the retina because the procedure is performed at a distance from the retina.

**Figure 1:**
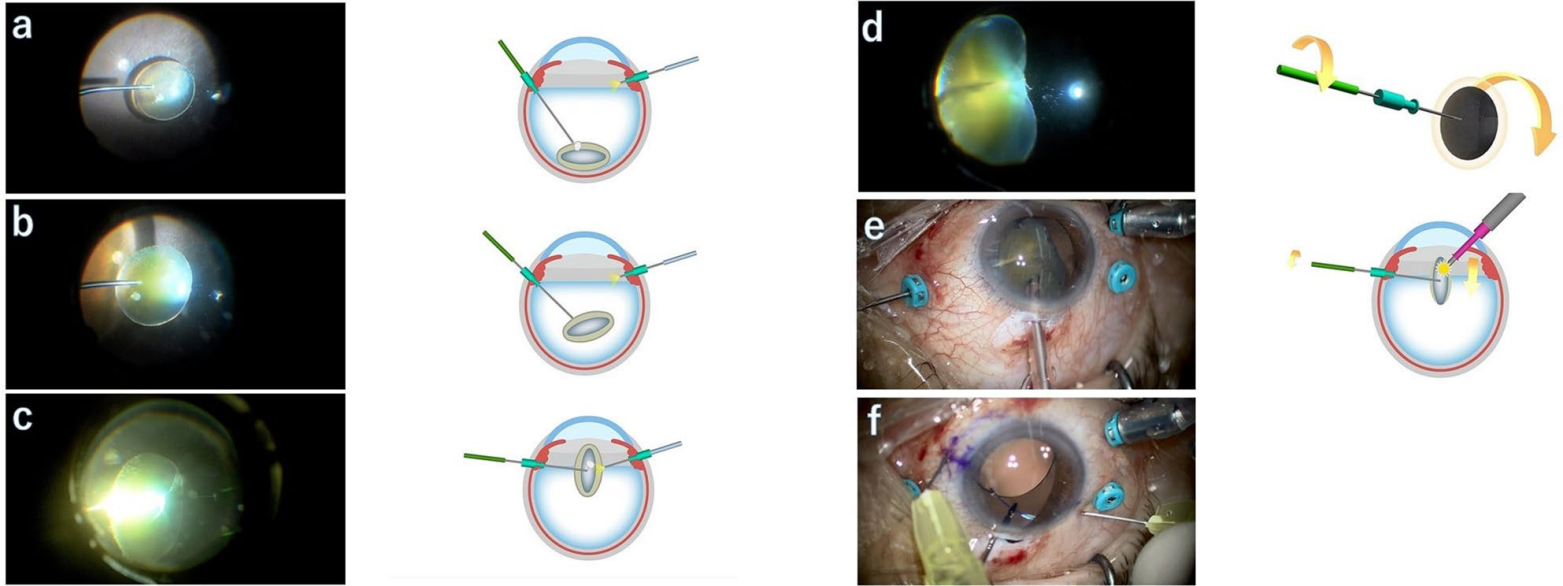
Kebab technique to remove the dropped nucleus of the lens. (Supplementary material S1). (**a**) A bipolar pencil touches the nucleus slightly, and surface coagulation is performed briefly. (**b**) The trapped nucleus is lifted up from the retina by the bipolar pencil. (**c**) The bipolar pencil recoagulates the lifted nucleus assisted by an endilluminator. (**d**) The bond becomes stronger, allowing the nucleus to be fully controlled by the bipolar pencil. (**e**) The lifted nucleus can then undergo phacoemulsification with complete control of its rotation. (**f**) After emulsifying the nucleus, the IOL is affixed by Yamane’s double-needle technique as necessary.

The first step of this procedure was to place the tip of the bipolar pencil close to the dropped nucleus of the lens (Fig. 1a). When the tip of the bipolar pencil lightly touched the nucleus slightly, surface coagulation was immediately performed for a brief time until the tip stuck to the center of the nucleus (Fig. 1b). The dropped nucleus of the lens affixed to the bipolar pencil was then gently lifted up from the retina.

The second step was to fix the nucleus more firmly to the tip of the bipolar pencil. The bonded bipolar pencil recoagulated the lifted nucleus assisted by an endoilluminator (Fig. 1c). When the tip of the bipolar pencil reached the center of the nucleus, the bond became stronger and the nucleus could be fully controlled by the bipolar pencil (Fig. 1d).

The third step was to subject the lifted nucleus to PEA. While lifting the nucleus to the anterior chamber, the phaco probe was inserted. We could rotate the bonded nucleus by turning the bipolar pencil without moving the phaco probe in the anterior chamber (Fig. 1e), allowing the nucleus to be shaved. If some nucleus fragments fell into the vitreous, they were removed after PEA with a 25-gauge vitreous cutter with a low cut rate, 500 to 1,000 cycles per minute. If necessary, the intraocular lens (IOL) was affixed by the Yamane double-needle intrascleral lens fixation technique (Fig. 1f), which is described in detail elsewhere^15^. In brief, an angled sclerotomy was made with a 30-gauge thin-wall needle, 1.5 mm from the limbus. The haptics were not cut before using the cautery device. The incision was expanded to 2.8 mm before IOL insertion. In all cases, eyes were checked again at the end of the operation for the presence of retinal detachment or residual lens.

Starting the day after surgery, topical 0.5% levofloxacin and 0.1% betamethasone eye drops were administered four times daily for one month and 0.1% bromfenac eye drops were administered twice daily for one month. We measured corneal endothelial cell density using Konan NSP 9900II specular microscopy (Konan Medical Inc., Nishinomiya, Japan) before and 1 month after surgery.

## Supplementary Information

Supplementary Information.
