# Supplementary material for: The kebab technique uses a bipolar pencil to retrieve a dropped nucleus of the lens via a small incision
Source: Sci Rep. 2021 Apr 12;11:7897. doi: 10.1038/s41598-021-87022-3 (PMC8041985; doi:10.1038/s41598-021-87022-3)
Supplement: Supplementary file 1 — Supplementary Information. [file 41598_2021_87022_MOESM1_ESM.docx]

**Original article**

The kebab technique uses a bipolar pencil to retrieve a dropped nucleus of the lens via a small incision

Hiroshi Aso※ • Harumasa Yokota • Hirotsugu Hanazaki • Satoru Yamagami •Taiji Nagaoka

All authors: Division of Ophthalmology, Department of Visual Sciences, Nihon University School of Medicine, 30-1 Oyaguchi-Kamicho, Itabashi-ku, Tokyo 173-8610, Japan

**operation video link:**

[
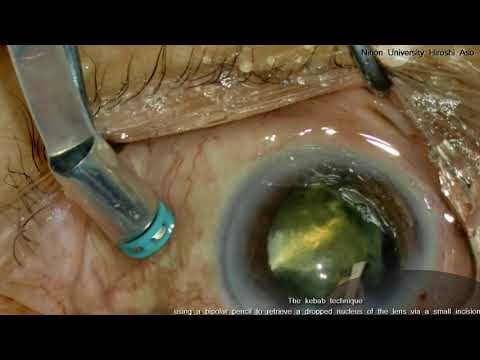
](https://www.youtube.com/embed/TvAfb8DZKg0?feature=oembed)

**https://youtu.be/TvAfb8DZKg0**
